# High Performance Clean Fracturing Fluid Using a New Tri-Cationic Surfactant

**DOI:** 10.3390/polym10050535

**Published:** 2018-05-16

**Authors:** Jinzhou Zhao, Jinming Fan, Jincheng Mao, Xiaojiang Yang, Heng Zhang, Wenlong Zhang

**Affiliations:** State Key Laboratory of Oil and Gas Reservoir Geology and Exploitation, Southwest Petroleum University, Chengdu 610500, China; 201621000788@stu.swpu.edu.cn (J.F.); 201621000749@stu.swpu.edu.cn (H.Z.); 201711000107@stu.swpu.edu.cn (W.Z.)

**Keywords:** tri-cationic surfactant, wormlike micelles, network, fracturing fluid

## Abstract

In order to improve the heat resistance of current clean fracturing fluids, a novel cationic surfactant (VES-T), composed of three single-chains and a spacer group, was designed and synthesized as thickener for the fluids. Various performances of such VES-T fluid in the presence of NaSal were evaluated carefully. Study of the rheological properties demonstrated that the fluids with varying concentrations (3–5 wt %) of VES-T have excellent thermal stabilities under ultra-high temperatures ranging from 140 to 180 °C. Until now, this is the highest temperature that the VES fracturing fluid could bear. The VES-T/NaSal fluid exhibited good viscoelasticity and proppant-suspending capability, which was attributed to the three-dimensional network formed by entangled wormlike micelles. Furthermore, the VES fracturing fluids can be completely gel broken by standard brines within 2 h. Thus, the VES-T synthesized in this work has a good prospect for utilization during the development of ultra-high temperature reservoirs.

## 1. Introduction

As an effective practice for increasing the rate of production of low-permeability reservoirs, hydraulic fracturing technology has been applied worldwide for decades [1]. Fracturing fluids have been utilized to generate artificial fractures in reservoir and transport proppant particles into the fracture, enhancing the conductivity of formation [2,3]. Polymer fluids such as polyacrylamide and guar gum have been widely used as thickeners for fracturing fluids. However, polymer fluids have the shortcoming of leaving non-removable residue in formation, which could plug the pore throats and cause serious formation damage [4,5]. In addition, due to the inherent high viscosity properties, the polymer-based fluids force the fracture to extend in height rather than in length [6,7]. Thus, other alternative fracturing fluids have gained great attention.

Being a substitute for conventional polymer fracturing fluid, the concept of viscoelastic surfactant (VES) fracturing fluid was first put forward by Schlumberger in 1997 [8]. Since then, VES fluids have been employed in the development of low-permeability gas and oil reservoirs for nearly 20 years [9]. Unlike polymer fracturing fluids, the sand suspension capability of the VES fluid depends largely on the elasticity instead of viscosity [10,11]. This unique property is rendered by the entangled wormlike micelles [12]. In general, the breaking of VES fracturing fluid after fracturing construction relies on two primary external conditions (reservoir conditions): (i) contact with hydrocarbons produced in the reservoir and (ii) dilution of reservoir brines [8,13]. Nevertheless, the VES fracturing fluid cannot break efficiently if the two reservoir conditions are not met in some cases, particularly in dry-gas reservoirs [14]. Usually, the internal breakers for VES fluid are used to address this problem. In comparison to traditional polymer fracturing fluid, VES fracturing fluid has a wide range of advantages, such as no insoluble residue, easy preparation in the well site, good sand-carrying capability, extremely low damage to formation, and low friction, etc. [15,16,17]. Furthermore, the VES foam is also applied as a treatment for ultralow-permeability reservoirs since it can reduce not only the interfacial tension but also the water consumption in the fracturing fluid [18]. In spite of the above mentioned advantages, the conventional VES fracturing fluid still has many deficiencies, which would limit its further application. For example, the traditional single head/single tail surfactants have high critical micelle concentrations (CMC) and exhibit poor surface-active behaviors. Thus, with these surfactants as thickener, the VES fracturing fluids are used at a much higher concentration than polymer fracturing fluids. Moreover, the capability of the known VES fracturing fluids to maintain high temperature and high shearing is quite limited. Meanwhile, with the rapid development of modern drilling technology, the exploitation and utilization of the ultra-high temperature oil and gas reservoirs attract tremendous attention. Accordingly, there is a great demand for VES fracturing fluids capable of resisting such high temperature.

Gemini surfactant is composed of two single head/single tail surfactants linked by a spacer between the head groups [19]. The spacer group restricts the electrostatic repulsion between hydrophilic groups through strong chemical bonds [20]. Hence, Gemini surfactants have higher surface activities and lower critical micelle concentration (CMC), exhibiting better rheological behaviors than single-chain surfactants [21,22]. Benefiting from the superiority of molecular design, the temperature resistant capacities of Gemini surfactant fracturing fluids are greatly promoted compared with single-chain surfactants. However, most of them still cannot have satisfactory performances (less than 150 °C). Therefore, the development of VES fracturing fluid with higher temperature resistance is the popular topic of research.

In view of the excellent properties brought by the combination of two single-chain monomeric surfactants, a question that emerges is whether a surfactant molecule consisting of more monomeric surfactants could have the better performance when compared to Gemini surfactants? In this work, a novel surfactant composed of three single head/single tail surfactants was synthesized using *N*,*N*′-Dimethyl-1,3-propanediamine, epichlorohydrin, and erucamidopropyl dimethylamine as raw materials. The performances of the fracturing fluid based on this surfactant, including surface activity, microstructure, viscoelasticity, thermo-shear resistance, sand suspending capability, and gel breaking property were investigated. In this way, a possible practical clean fracturing fluid could be developed for the various oilfields. It is noteworthy that, to our knowledge, this novel VES fracturing fluid could bear the highest temperature (up to 180 °C) till now, which is potentially useful for the exploration of deep or super deep reservoirs.

## 2. Materials and Experiments

### 2.1. Materials

The product named as VES-T was synthesized as follows. Erucamidopropyl dimethylamine was provided by Shanghai Winsono New Material Tech Co., Ltd. (Shanghai, China). *N*,*N*′-Dimethyl-1,3-propanediamine, epichlorohydrin, ethanol, concentrated hydrochloric acid (HCl), n-hexane, acetone, ethyl acetate, potassium chloride (KCl), and sodium salicylate (NaSal) were obtained from Sinopharm Chemical Reagent Co., Ltd. (Shanghai, China). Deionized water was used in all measurements.

### 2.2. Synthesis of VES-T

#### 2.2.1. Synthesis of Intermediate

**Scheme 1:**
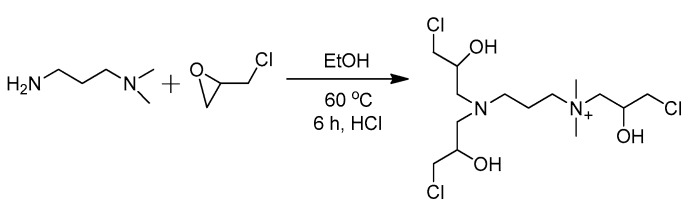
Synthesis of intermediate.

Ethanol (50 mL) and *N*,*N*′-dimethyl-1,3-propanediamine (6.13 g, 0.06 mol) were added into a round-bottom flask. Epichlorohydrin (27.76 g, 0.3 mol) was then added dropwise to this stirring mixture at 25 °C. Subsequently, concentrated hydrochloric acid (HCl) (6 g, 0.06 mol) was added to provide hydrogen atoms for this reaction. Then, the mixture was stirred vigorously under reflux at 60 °C for 6 h (Scheme 1). The solvent ethanol was removed by vacuum rotary evaporator. The intermediate was extracted for three times with a mixture of deionized water and *n*-hexane (1:1) to remove residual epichlorohydrin.

#### 2.2.2. Synthesis of VES-T

**Scheme 2:**
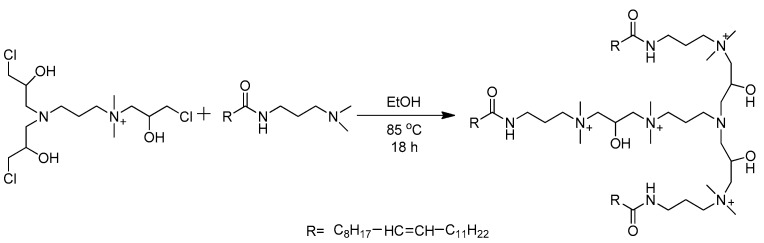
Synthesis of VES-T.

The intermediate (24.97 g, 0.06 mol) was dissolved in ethanol (60 mL). Then, erucamidopropyl dimethylamine (76.14 g, 0.18 mol) was added into the round-bottom flask. During the reaction process, the mixture was stirred vigorously under reflux at 85 °C for 18 h (Scheme 2). Next, ethanol was evaporated under reduced pressure. The crude product obtained was repeatedly washed with acetone and then recrystallized from a mixture of ethyl acetate and ethanol (7:1) for three times to yield the pure VES-T.

### 2.3. Measurement

**Figure 1:**
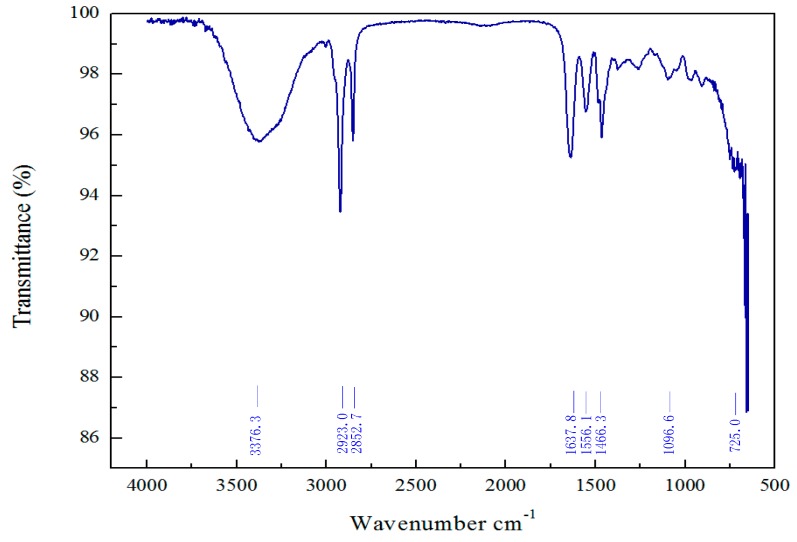
Fourier-transform infrared spectroscopy (FT-IR) spectra of VES-T.

**Figure 2:**
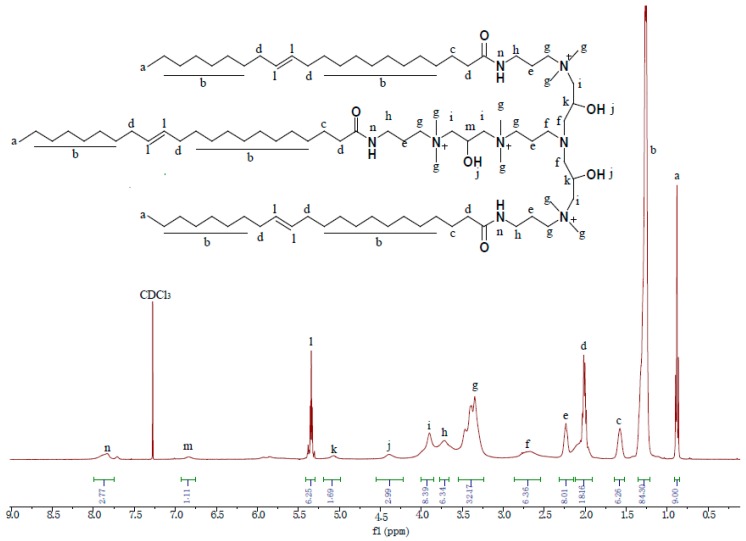
^1^H NMR spectrum of VES-T in CDCl_3_.

The ^1^H nuclear magnetic resonance (^1^H NMR) spectral analysis of VES-T was performed using a BRUKER AVANCE III HD 400 MHz spectrometer (Bruker, Karlsruhe, Germany). Fourier-transform infrared (FT-IR) spectrum was obtained on a Nicolet MAGNA IR 560 ESP FT-IR spectrometer (Nicolet, Madison, WI, USA). The FT-IR and ^1^H NMR spectroscopic results are shown in Figure 1 and Figure 2.

Surface tension measurement of VES-T was conducted using KRUSS DSA30S tensiometer (KRUSS, Hamburg, Germany). Scanning electron microscope (SEM) micrographs were obtained from a Quanta 450 scanning SEM (FEI, Hillsboro, OR, USA). The samples were prepared by freezing a drop of VES-T fracturing fluid with liquid nitrogen (−185 °C), where the microstructure of the solutions could be retained. Subsequently, the frozen samples were dried and then coated with gold. Rheological properties were evaluated using a HAAKE MARS III Rheometer (Thermo Scientific, Munich, Germany). Viscoelasticity was determined by Anton Paar physical MCR 301 Rotational Rheometer (Anton Paar, Graz, Austria).

#### Fracturing Fluid Preparation

The VES fracturing fluid mainly contains two components, wherein the VES acts as a thickener, and counter-ion additive screens the electrostatic repulsion between the hydrophilic head groups and promotes the growth of wormlike micelles. The fracturing fluids with varying concentrations (3 and 5 wt %) of VES-T were prepared to evaluate their rheological properties, viscoelasticity, sand-carrying abilities, and gel breaking properties. The concentrations of NaSal in the two fracturing fluids were 1 and 1.2 wt %, respectively. Additionally, the fluid sample with KCl as counter-ion was also prepared to investigate its effect on the formation of the network. The samples were centrifuged before tests to remove the air bubbles.

## 3. Results and Discussion

### 3.1. Structural Characterization of VES-T

The structure of VES-T was confirmed by FT-IR and ^1^H NMR. Figure 1 shows the FT-IR spectrum of VES-T. The absorption peak resulting from –O–H is presented at 3376.3 cm^−1^ and the absorption bands corresponding to the CH_3_ and CH_2_ stretch on the acylamino group are found at 2923.0 cm^−1^ and 2852.7 cm^−1^, respectively. The absorption peak at 1637.8 cm^−1^ is due to the C=O stretching vibration absorptions. The N–H stretching peak appears at 1556.1 cm^−1^. Moreover, the peak at 1466.3 cm^−1^ corresponds to C–N stretching vibration.

Figure 2 is about the ^1^H NMR (400 MHz, CDCl_3_) for VES-T: 0.88 (t, 9H, CH_3_), 1.26 (m, 84H, CH_2_), 1.58 (s, 6H, COCCH_2_), 2.03–1.98 (m, 18H, C=CCH_2_, COCH_2_), 2.23 (m, 8H, N^+^CCH_2_), 2.70 (s, 6H, NCH_2_), 3.47–3.35 (m, 32H, N^+^CH_3_, N^+^CH_2_), 3.72 (s, 6H, CONCH_2_), 3.90 (s, 8H, CN^+^CH_2_), 4.39 (s, 3H, COH), 5.07 (s, 2H, N^+^CCH), 5.38–5.30 (m, 6H, HC=CH), 6.83 (s, 1H, N^+^CCH), 7.83 (m, 3H, CONH). Thus, the desired product VES-T was successfully synthesized.

### 3.2. Surface Tension Measurement

**Figure 3:**
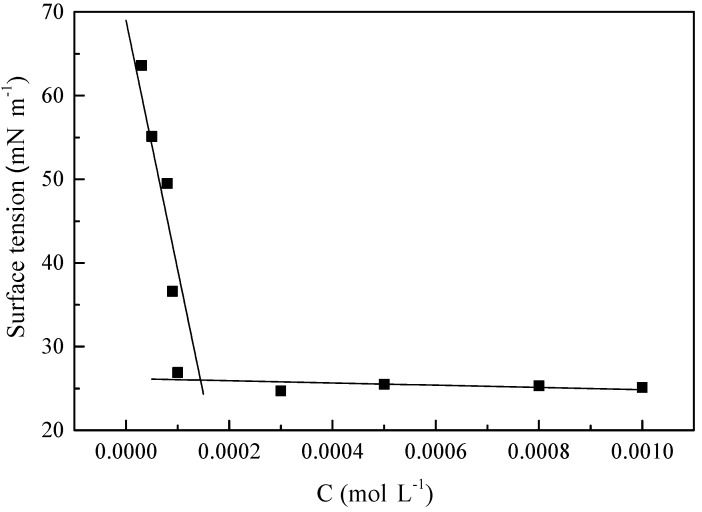
Surface tension plot for VES-T tri-cationic surfactant.

As mentioned earlier, the surface tension of VES-T was measured at 25 °C using a KRUSS DSA30S tensiometer. The measurement process continued until the surface tension value obtained was almost constant. Shown in Figure 3 is the variation of surface tension versus the concentration of VES-T. The variation tendency of surface tension could be described with two straight lines. The surface tension decreases with the increasing concentration of VES-T, and then reaches a clear inflection point, where the intersection of the two lines is the CMC. The surface tension at CMC is recorded as γCMC.

As shown in Figure 3, the CMC of the corresponding product VES-T is 1.44 × 10^−4^ mol/L and the γCMC is 25.98 mN/m. Akram et al. [19] designed a Gemini surfactant (16-E2-16), whose CMC and γCMC value are 1.2 × 10^−3^ mol/L and 42.6 mN/m, respectively. Apparently, the CMC and γCMC of VES-T are both smaller than that of 16-E2-16, which is consistent with the previous assumption that the surfactant composed of three hydrophobic groups has a better performance than general Gemini surfactants.

### 3.3. Scanning Electron Microscopy (SEM)

As SEM visualizes the micellar morphology directly, it was employed to investigate the microstructure of the VES-T fracturing fluid, which could also associate the microstructure with macroscopic properties, such as the rheology and viscoelasticity of the VES-T fracturing fluid [23]. According to Candau et al. [20], the self-aggregation and entanglement of surfactant micelles largely depend on the nature and concentration of salts. The salts would help transform spherical micelles into rod-like or wormlike micelles [24,25].

**Figure 4:**
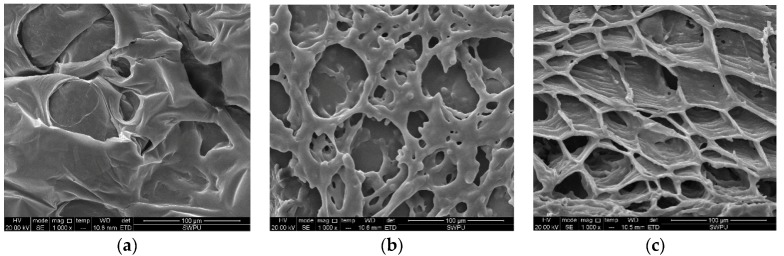
The Scanning electron microscopy (SEM) images of (**a**) VES-T solution without any counter-ion (served as a blank control); (**b**) VES-T solution with NaSal; (**c**) VES-T solution with KCl. The cross-linked networks could be observed in both (**b**) and (**c**) in comparison to (**a**).

For this reason, the SEM measurements of the VES-T fracturing fluids without any counter-ion, with NaSal and KCl were conducted using a Quanta 450 scanning SEM (FEI, Hillsboro, OR, USA), respectively. The results are shown in Figure 4a–c. Compared with Figure 4a, b and c both show cross-linked networks composed of entangled wormlike micelles. This result confirms that the presences of counter-ions (NaSal and KCl) greatly assist the formation of 3D networks, and the shielding effects by overhead counter-ions are remarkable. The viscoelastic and rheological characters of the VES-T fracturing fluid can be explained as the formation of the 3D network.

### 3.4. Resistance to High Temperature and High Shear

**Figure 5:**
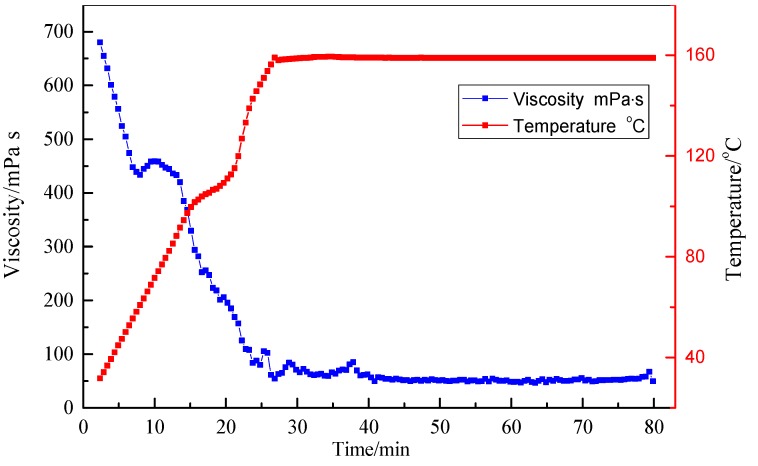
Shear resistance of 5 wt % VES-T with 1.4 wt % KCl fracturing fluid at 160 °C, 170 s^−1^.

**Figure 6:**
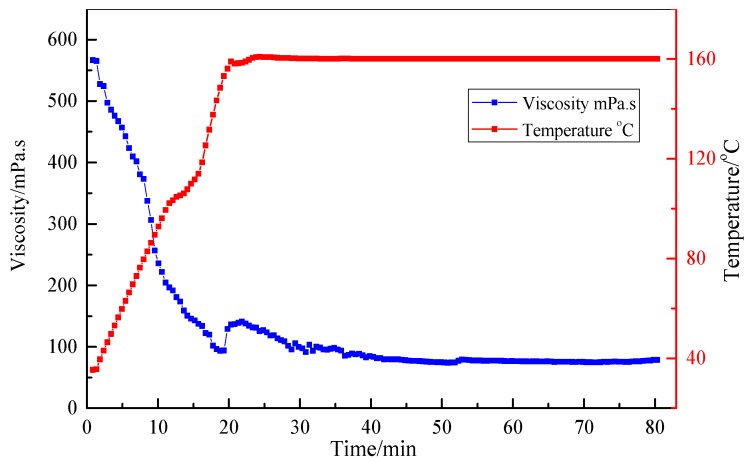
Shear resistance of 5 wt % VES-T with 1.2 wt % NaSal fracturing fluid at 160 °C, 170 s^−1^.

Both KCl and NaSal have significant effects on the formation of entangled wormlike micelles according to the SEM morphologies of Figure 4a–c. For the purpose of investigating the influence of the counter-ions on the macroscopic rheological properties of the VES-T fluids, the thermal-stability tests for the VES-T fluids with KCl and NaSal were performed at 160 °C and at the constant shear rate of 170 s^−1^ for 80 min. The results are shown in Figure 5 and Figure 6. As depicted in Figure 5, the viscosity of the solution prepared with 5 wt % VES-T and 1.4 wt % KCl first decreases to 433 mPa·s, and then increases to 458 mPa·s. Subsequently, the viscosity decreases again and finally maintains at about 50 mPa·s. Similarly, for a solution consisting of 5 wt % VES-T and 1.2 wt % NaSal, the viscosity of fluid decreases sharply with the increase of testing temperature, and then maintains at about 78 mPa·s. The results indicate that NaSal has a superior performance in enhancing the thermal stability of the system compared with KCl.

**Figure 7:**
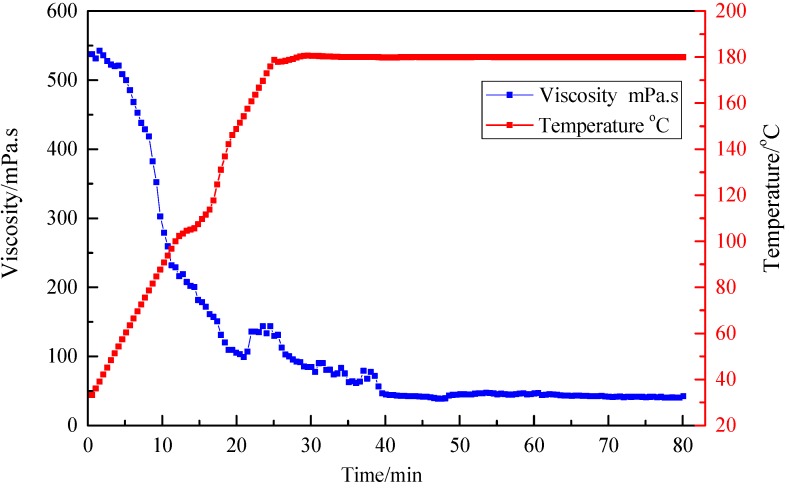
Shear resistance of 5 wt % VES-T with 1.2 wt % NaSal fracturing fluid at 180 °C, 170 s^−1^.

In view of the excellent rheological property that VES-T/NaSal solution exhibited at 160 °C, the thermal stability test was further conducted at 180 °C. As depicted in Figure 7, the VES-T/NaSal solution displays a similar rheological behavior to that at 160 °C, and the viscosity is constant at about 42 mPa·s till the end of test. Obviously, NaSal is more suitable to be the counter-ion of the system.

**Figure 8:**
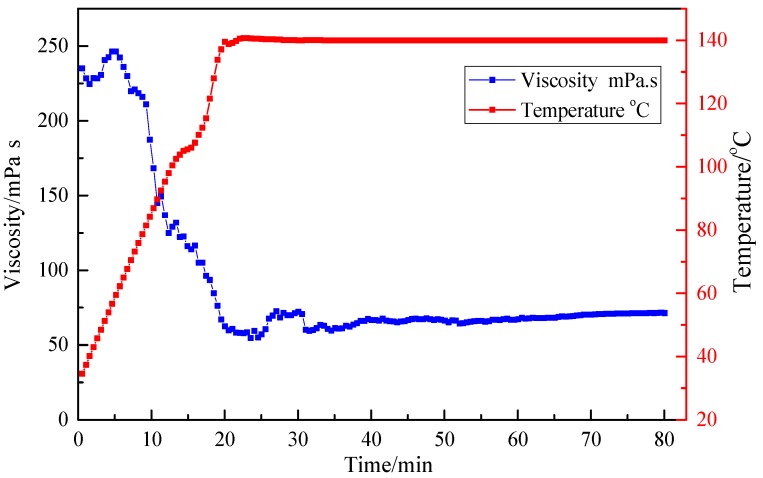
Shear resistance of 3 wt % VES-T with 1 wt % NaSal fracturing fluid at 140 °C, 170 s^−1^.

To reduce the cost, the concentration of VES-T was reduced to 3 wt %, and the rheological property of the system was evaluated at 140 °C. As shown in Figure 8, the viscosity of the 3 wt % VES-T/1 wt % NaSal solution remains at 70 mPa·s, which could meet the viscosity requirement (>25 mPa·s) for VES fracturing fluid according to industry standard. The thermal-stability testing results demonstrate that the VES-T fracturing fluids with the above formula could have good performance in the development of reservoirs under ultra-high temperature.

**Figure 9:**
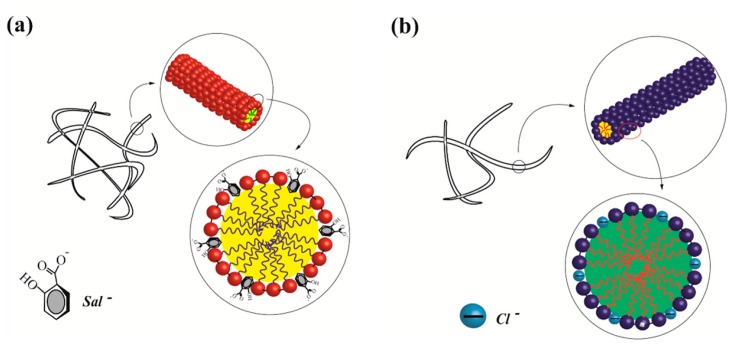
(**a**) Schematic diagram for Sal^−^ to affect micelle shape transition; (**b**) Schematic diagram for Cl^−^ to affect micelle shape transition.

Some studies reported that both chlorides and aromatic compounds, such as KCl and NaSal, could induce micelle morphology transition for cationic surfactant from spherical to wormlike [26,27,28,29,30,31,32,33,34]. However, there are some differences between the effects of KCl and NaSal. Chlorine anions (Cl^−^) are adsorbed on the surface of the cationic hydrophilic head group to form an electric double layer around the micelle shell, which screens the electrostatic repulsion. Alternatively, in addition to the similar surface adsorption of Cl^−^, the aromatic anion (Sal^−^) penetrates the surface of the micelle, whose aromatic nucleus has a strong hydrophobic interaction with the surfactant, increasing the surfactant-packing parameter by efficiently decreasing the area per surfactant monomer (*a*_0_) [28,29,31]. Therefore, Sal^−^ is a far more effective driving force to the growth of the worm-like micelles. The mechanism by which Sal^−^ and Cl^−^ affect the micelle shape transition is schematically illustrated in Figure 9a,b, respectively. Additionally, the sphere-rod transition of micelles generally occurs with the lower addition of Sal^−^ than Cl^−^ [28,30,32,33,34], which agrees well with the experimental results.

### 3.5. Viscoelasticity Evaluation

**Figure 10:**
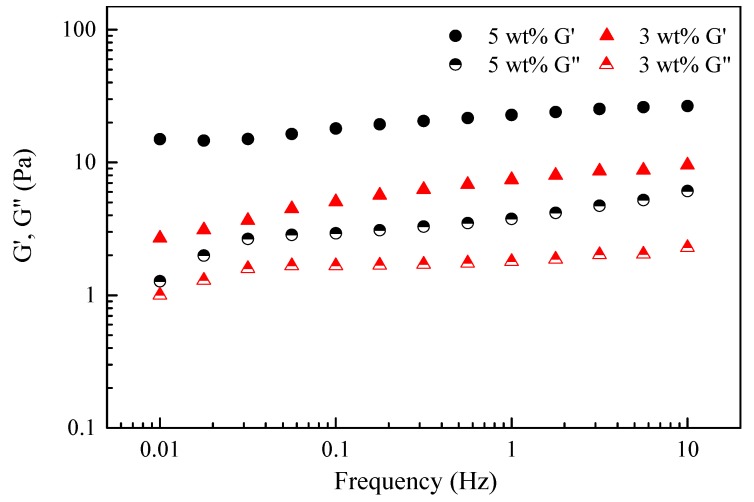
Storage modulus *G*′ and loss modulus *G*″ varying as a function of frequency for the two (3 wt % and 5 wt %) VES-T samples at 25 °C. G′ is greater than *G*″ throughout the test in both the two samples. *G*′ and *G*″ of the 5 wt % VES-T sample are greater than that of the 3 wt % VES-T sample.

Compared with traditional viscous fluids, the proppant suspension of VES fracturing fluid is strongly affected by both viscosity and elasticity, that is, the viscoelasticity. Viscoelasticity is an essential property to evaluate the performance of VES fracturing fluid, which is attributed to the formation of a 3D network. Therefore, the viscoelasticity of the VES-T solution in the presence of NaSal was measured by an Anton Paar physical MCR 301 Rotational Rheometer, as shown in Figure 10.

In each solution, storage modulus *G*′ and loss modulus *G*″ both slightly increase with the increase of frequency during the range of frequency scanning, and storage modulus *G*′ is greater than loss modulus G″ throughout the test. Accordingly, the two solutions both exhibit typical elastic behaviors, indicating the formation of entangled wormlike micelles. Moreover, it is apparent that the storage modulus G′ and loss modulus *G*″ of the VES solution both increase with the increasing concentration of VES-T from 3 wt % to 5 wt %, which demonstrates that the increasing VES-T concentration in the presence of NaSal augments the entanglements. The increase of VES concentration results in a more obvious viscoelasticity of the VES fracturing fluid [35].

### 3.6. Proppant Suspension Measurement

Good sand-carrying capability is essential to the fracturing fluid, which allows it to carry proppant evenly into the fractures [36]. If the sand-carrying capability of the fracturing fluid is poor, the proppant will fall rapidly and cannot be transported into deep fractures in the reservoir [37]. The static proppant settling tests for the VES-T fracturing fluids with abovementioned formula were carried out at 80 °C to evaluate the sand-carrying capabilities. 50 mL of VES fluid and 10 g of ceramics of mesh size 20/40 were mixed uniformly and then poured into a 50 mL graduated cylinder. After 120 min under the experimental conditions, almost no ceramics settled in 5 wt % VES-T/1.2 wt % NaSal fluid. For the fluid sample with 3 wt % VES-T, a small amount of proppant settled and the settling velocity was 0.007 mm/s. The proppant settlement phenomenon was more obvious in fluid with lower VES-T concentration. When the experiments were conducted at 25 °C, few proppant settled in both the two fluid samples after 2 d. Thus, the fracturing fluids with above formula could meet the demand for suspending proppant during the fracturing construction. The proppant suspension of the VES fracturing fluid depends on both viscosity and elasticity of the system, which is quite different from that of the polymer and guar gum fracturing fluids.

### 3.7. Gel Breaking Test

The gel breaking process must be completed rapidly and thoroughly after fracturing construction to minimize the damage to the reservoir. Generally, the fracturing fluid is considered to be completely broken once the viscosity of the breaking fluid is less than 5 mPa·s. VES fracturing fluid usually relies on reservoir conditions such as standard brines and hydrocarbons to achieve the purpose of gel breaking. Hence, the VES-T fracturing fluids with the above formula were mixed separately with different proportions of standard brine and kerosene at 90 °C. As shown in Table 1 and Table 2, when the weight ratios are higher than 1:4 (standard brine to 3 wt % VES fluid) and 1:10 (standard brine to 5 wt % VES fluid), the fracturing fluids are completely broken and the viscosities of the breaking fluids are both less than 5 mPa·s within 2 h. Moreover, it is found that the VES-T fracturing fluid breaks quicker with the higher volume ratio of brine. For the reservoir hydrocarbon condition, when 30 wt % of kerosene is added, the viscosities of fluids with above formula are below 3 mPa·s within 30 min.

These results confirm that the gel breaking properties of the VES fluids of the above formula are sufficient to fulfill the requirements for flowback treatment of fracturing fluid.

### 3.8. General Description

The tri-cationic surfactant (VES-T) developed as a thickener for clean fracturing fluid was synthesized in a simple way with high yield (>96%). As far as we know, this is the first example of tri-cationic surfactant for the fracturing fluid. The main feedstock used in synthesis of the VES-T is erucic acid, which comes from the rapeseed. Thus, it is environmental friendly. The heat resistance of the VES-T fracturing fluid far exceeds that of the majority of the existing VES fracturing fluids. For instance, the 2% erucyldimethyl amidopropyl amine oxide (EMAO) solution cannot have good performance over 90 °C [38]. A VES-HT fracturing fluid could only fulfill the requirement of 275 °F (135 °C) according to Schlumberger [39]. Nevertheless, compared with the generally used single-chain and Gemini surfactants, the molecule of VES-T consists of more monomeric surfactants, and the price of erucic acid is higher than commonly used raw materials such as oleic acid, which would slightly increase the cost. However, if the rapeseed could be directly employed as the starting material, the cost will be reduced sharply. Work is ongoing in our lab to further optimize the formula to reduce the amount of VES.

## 4. Conclusions

A novel surfactant consisting of three single head/single tail surfactants was synthesized as thickener for VES fracturing fluid. The product was characterized by ^1^H NMR and FT-IR. The two optimized formula of VES fracturing fluids were determined to be 5 wt % VES-T + 1.2 wt % NaSal and 3 wt % VES-T + 1 wt % NaSal, and both of them have excellent performances in various tests.

(1). The surface tension measurement data confirm that the combination of three single-chain surfactants leads to a lower CMC and higher surface activity than the conventional Gemini surfactants.
(2). Microstructure study suggests that the organic (NaSal) and inorganic (KCl) salts both have significant effects on the micellar transition from spherical micelles to wormlike micelles.
(3). The fracturing fluids with the two concentrations (3 and 5 wt %) of VES-T both exhibit excellent rheological properties under high temperature. Especially, the viscosity of fluid sample containing 5 wt % VES-T and 1.2 wt % NaSal maintains at about 42 mPa·s at 180 °C, which is the highest temperature recorded till now.
(4). Viscoelasticity evaluation shows typical elastic properties of the two fracturing fluids. Additionally, the storage modulus *G*′ and loss modulus *G*″ of the VES solution both increased with the increasing concentration of VES-T, indicating a more obvious viscoelasticity of the VES fracturing fluid. In other words, a higher VES concentration leads to a stronger network structure.

The two developed VES fracturing fluids also have many other advantages, such as strong sand suspending abilities and easy gel breaking, etc. These benefits demonstrate that the synthesized product can be a promising application in the development of gas and oil reservoirs with ultra-high temperature.

## Figures and Tables

**Table 1:** 3 wt % viscoelastic surfactant (VES) fluid gel breaking at different brine ratios.

Ratio (Standard Brine to Fluid)	Breaking Time (h)	Viscosity of Breaking Fluid (mPa·s)
1:5	2.3	4.9
1:4	1.8	4.1
1:3	1.1	3.1

**Table 2:** 5 wt % VES fluid gel breaking at different brine ratios.

Ratio (Standard Brine to Fluid)	Breaking Time (h)	Viscosity of Breaking Fluid (mPa·s)
1:12	2.6	4.3
1:10	1.9	3.8
1:8	1.3	2.9
